# Tuberculosis of Bones and Joints*Read before Atlanta Society of Medicine. (Subject selected by President.)

**Published:** 1898-10

**Authors:** Floyd W. McRae

**Affiliations:** Atlanta, Ga.; Professor of Gastro-Intestinal, Rectal and Clinical Surgery Atlanta College of Physicians and Surgeons; Elected to deliver Address on Surgery before American Medical Association, 1899; Ex-Secretary Section on Surgery and Anatomy of American Medical Association; Ex-Vice-President Southern Surgical and Gynecological Association and Tri-State Medical Association of Georgia, Alabama and Tennessee; Ex-Secretary and President Atlanta Society of Medicine


ATLANTA

Medical and Surgical Journal.

Vol. XV. OCTOBER, 1898. No. 8.

DUNBAR ROY, A.B., M.D., EDITOR. M. B. HUTCHINS, M.D., BUSINESS MANAGER.

### ORIGINAL COMMUNICATIONS.

### TUBERCULOSIS OF BONES AND JOINTS.*

By FLOYD W. McRAE, M.D., Atlanta, Ga.

Professor of Gastro-Intestinal, Rectal and Clinical Surgery Atlanta College of Physicians and Surgeons; Elected to deliver Address on Surgery before American Medical Association, 1899; Ex-Secretary Section on Surgery and Anatomy of American Medical Association; Ex-Vice-President Southern Surgical and Gynecological Association and Tri-State Medical Association of Georgia, Alabama and Tennessee; Ex-Secretary and President Atlanta Society of Medicine.

I shall only briefly review some of the most important facts bearing upon this very interesting class of diseases.

For centuries these chronic inflammatory conditions of bones and joints have been recognized as local manifestations of a constitutional condition. There are a few very able surgeons who are of the opinion that injuries to the bones and joints are a predisposing cause. I shall not attempt to discuss the difference between strumous and tubercular bone diseases, because, in my opinion, they are the same. I shall briefly submit for your consideration some of the reasons which convince me of the tuberculous character of

*Read before Atlanta Society of Medicine. (Subject selected by President.)

these affections. In doing so I shall follow Senn's work on "Tuberculosis of Bones and Joints."

There are quite a number of cases on record which show clearly that these conditions have been the result of direct infection, and the following case reported by Verneuil, of a "student who injured the fold of the nail of his right finger at a post-mortem, with the result of causing a local tuberculosis of the skin. This was treated in various ways without permanent improvement, and after treatment of three years there was still a tubercular ulcer on the finger and abscess on the back of the hand. This abscess was opened and the ring finger was amputated, but chronic abscesses continued to form, and the patient died six years after the injury of spinal meningitis, due to suppuration in connection with tubercular disease of the vertebra." (Senn.)

*The numerous cases of inoculation reported by various observers.*—The inoculations are almost uniformly productive of either general or local tuberculosis, according to the amount of the material used and the point of inoculation. It seems to make very little difference whether tubercle bacilli are injected directly, granulation tissue implanted, or other tuberculous material used, there is undoubtedly a direct relationship existing between the amount of infection and the severity of the disease. Animals receiving a large number of bacilli directly in the circulation soon die of general miliary tuberculosis, while a small amount of tubercular matter embedded in the tissues produces, primarily, local manifestations, with subsequent general infection. It is further shown by these experiments that it is necessary to inject a definite amount of tuberculous matter in order to produce any reaction at all.

*Bone and joint tuberculosis have been produced in the lower animals by direct inoculation.*—"Stuttgart, in 1880, studied the localization of the tubercular virus experimentally in the same manner as others have studied the localization of pus microbes. He inoculated animals with the products of tubercular inflammation; subsequently produced contusions and sprains of joints, and observed that localization usually occurred at the seat of the injury. If the tuberculous virus was introduced by inhalation, the same typical lesions occurred in the injured joint as when injection was made more directly. In all cases the products of the local lesion correspond with the character of the material introduced through some remote point." (Senn.)

*The frequent association of bone and joint tuberculosis with tuberculosis of other organs.*—"Billroth and Menzel found, on searching the post-mortem records at Vienna for a period of fifty years (1817 to 1867), that there had been 20,106 cases of caries of bones and joints, and of these more than half were complicated with tuberculosis of the internal organs. Neumeister has collected 438 cases from the Wurzburg Clinic and other sources, with 60, or 15 per cent., of deaths from acute tuberculosis. Willemer had ascertained from some statistics which he collected and studied, that, in case of chronic affection of the knee-joint, one per cent. of the patients die of tuberculosis during the first year of the disease, seven per cent. during the second, six per cent. during the third, making a mortality of fourteen per cent. from acute tuberculosis within three years.

"Konig states that in only 21 per cent. of all cases of joint tuberculosis is the disease confined to the joints." (Senn.)

*The microscopical structure of the diseased tissue and the caseation of the inflammatory products.*—The most careful observers report series after series of cases where they have not failed in a single instance to find tubercle bacilli, though the search is frequently tedious and long before they are discovered. Bacilli are found in the pus, in the caseous material, in the granulation tissue, etc.

Another proof which Senn presents is the reaction to tuberculin. There are a few interesting pathological facts to which I wish to call your attention. One of the most interesting and important to a thorough understanding of the subject is the scant blood supply of the tuberculous nodule. Whether the beginning of the process is in the blood or in the connective tissue, there is, sooner or later, a complete destruction of the blood-vessels in the affected area. When the point of infection is within a blood-vessel there is immediate anemia, the result of a tubercular thrombus in the vessel. Where the infection occurs outside of the vessel, granulation tissue forms, and there is a gradual growth of the tubercular nodule, separating the vessels and finally so compressing them as to completely shut off the circulation through them. This accounts for the production of the "wedge-shaped sequestra" so frequently observed in the extremities of long bones. The tubercular nodules enlarge by the development of granulation tissue. The tubercle bacillus attacks more frequently connective tissue than any other structure, though its ravages are not confined by any means to connective tissue.

One of the most interesting manifestations of the tubercular process is caseation, a condition which is not well understood by pathologists, but which seems to follow coagulative necrosis resulting from tubercular inflammation. Very frequently small masses of caseous material are found which have existed for years without producing either local or general symptoms, and not infrequently bone and joint lesions seem to have had their origin in these caseous masses. Tubercle bacilli may remain for years embedded in this caseous material without producing symptoms. When, however, as a result of injury or diminished resistance of the surrounding tissue from any cause, there is liquefaction of this material, the bacilli are taken up by the absorbents and transported to more or less distant points.

The tubercular abscess, or cold abscess, as it is called, contains a kind of emulsion composed of liquefied tubercular material, and is never actively inflammatory, unless infected with either streptococci or staphylococci. A comprehensive review of the various manifestations of tubercular ostitis, properly osteomyelitis, would be interesting had we sufficient time at our disposal. Though these conditions are the result of a general dyscrasia, traumatism is often the exciting cause. The trauma simply diminishes local resistance, and there develops a local tuberculosis, though the point of entrance of the bacilli may have been either the lungs or the alimentary canal. The bacilli circulated harmlessly through the system until a point of diminished resistance was reached.

The diagnosis of tubercular bone disease is difficult in obscure cases. Pain is always present in tuberculous joints, though usually not acute. The pain is not infrequently reflected to a more or less distant point. For instance, pain in the knee in diseases of the hip-joint, or persistent pain in the chest or abdomen in diseases of the vertebra. There is always a point of extreme tenderness corresponding to the carious point in the bone. Swelling is more or less marked, though sometimes only slight. There is never redness or discoloration of the skin, unless there is mixed infection with abscess formation, or the skin is involved in the tuberculous process. Muscular atrophy is marked.

The prognosis in these cases of bone and joint disease is, in the majority of instances, favorable, provided patients are seen early and can be controlled and given proper treatment. The treatment is both local and general. Every effort should be made to bring about a general improvement in the health: tonics, cod-liver oil, nutritious diet, sunshine, exercise in the open air, and every agency which tends to the upbuilding of the general health, are of value in the treatment of these affections. I shall, however, in this paper devote my remarks to local treatment, as the subject of general treatment has recently been gone over very thoroughly by one of my colleagues before this society. The local measures which may be employed are numerous, and possess more or less value according to existing conditions. Of first importance in all cases is the great principle of rest. This may be brought about by the numerous mechanical devices which are employed, or by putting patient to bed and enjoining strict recumbency. Where rest to the inflamed bone or joint can be procured, and the patient be allowed at the same time free exercise in the open air, the best results are to be expected. When it is necessary to put the patient to bed, it is a delicate question to decide just how long he or she shall be kept there, and there is quite a divergence of opinion on this subject. Where there is extensive destruction of tissue in the joint, with or without active inflammatory conditions, more radical measures are indicated: resection, removal of sequestra, and amputation even, are occasionally called for.

Cases seen early should be immediately fitted with proper apparatus to secure rest to the inflamed joint, and such general treatment instituted as indicated. Counter-irritation, and especially the actual cautery, gives good results in properly selected cases. This treament should be persevered in for months or years, as demanded. Special modifications may be necessary from time to time, but the general plan above indicated should be persisted in until a cure is effected, or complications arise which demand more radical measures. When there is marked accumulation of fluid in a joint, or a large cold abscess has formed, the fluid should be drawn off, the cavity washed out carefully with boric acid or other mild antiseptic solution, and a ten per cent. emulsion of iodoform and glycerin or olive-oil injected and allowed to remain. This operation should be done under the strictest antiseptic precautions.

Infection with streptococci or staphylococci converts a cold abscess into a pus cavity, and adds to a tubercular joint the dangers of an acute suppurative inflammation. After infection with either streptococci or staphylococci, with subsequent suppuration, free incision, careful cleansing and free drainage must be resorted to or serious results soon follow. In advanced cases resections of joints are frequently called for and amputations are sometimes necessary in order to save the life of the patient. Where there is carious bone outside of joints, it should be removed whether the amount be large or small. Extensive destruction can often be prevented by prompt incision through all the soft parts, including the periosteum. All sequestra should be removed, but healthy bone and periosteum should be preserved.

I have several times removed the whole shaft of the tibia, leaving new bone and periosteum, with most excellent results.

All tubercular cavities should be most carefully cleansed by curetting, wiping and washing, so as to insure the thorough removal of all tuberculous material. As the tubercle bacilli are found in the granulation tissue, it also should be removed.

The final success of the operation will depend upon the thoroughness with which these measures have been carried out and the general condition of the patient.

63½ *Whitehall St.*

---

Corsets Prohibited.—The Russian Minister of Public Instruction has issued a decree, prohibiting the use of corsets by women.—*Ex.*

